# Docetaxel-Loaded Chitosan Microspheres as a Lung Targeted Drug Delivery System: *In Vitro* and *in Vivo* Evaluation

**DOI:** 10.3390/ijms15033519

**Published:** 2014-02-26

**Authors:** Hao Wang, Yongdong Xu, Xiao Zhou

**Affiliations:** 1 Department of Thoracic surgery, Shanghai Pulmonary Hospital, Tongji University School of Medicine, 507 Zheng Min Road, Yangpu District, Shanghai 200433, China; E-Mail: wanghao21384313@163.com; 2 Department of Thoracic surgery, Shanghai Pudong Hospital, Fudan University Pudong Medical Center, 2800 Gong Wei Road, Hui Nan Town, Pudong, Shanghai 201399, China; E-Mail: xyd927927@163.com

**Keywords:** docetaxel, microspheres, release, pharmacokinetics, biodistribution

## Abstract

The aim of this study was to prepare docetaxel-loaded chitosan microspheres and to evaluate their *in vitro* and *in vivo* characteristics. Glutaraldehyde crosslinked microspheres were prepared using a water-in-oil emulsification method, and characterized in terms of the morphological examination, particle size distribution, encapsulation ratio, drug-loading coefficient and *in vitro* release. Pharmacokinetics and biodistribution studies were used to evaluate that microspheres have more advantage than the conventional formulations. The emulsion crosslinking method was simple to prepare microspheres and easy to scale up. The formed microspheres were spherical in shape, with a smooth surface and the size was uniform (9.6 ± 0.8 μm); the encapsulation efficiency and drug loading of prepared microspheres were 88.1% ± 3.5% and 18.7% ± 1.2%, respectively. *In vitro* release indicated that the DTX microspheres had a well-sustained release efficacy and *in vivo* studies showed that the microspheres were found to release the drug to a maximum extent in the target tissue (lung). The prepared microspheres were found to possess suitable physico-chemical properties and the particle size range. The sustained release of DTX from microspheres revealed its applicability as drug delivery system to minimize the exposure of healthy tissues while increasing the accumulation of therapeutic drug in target sites.

## 1. Introduction

**Figure 1.**
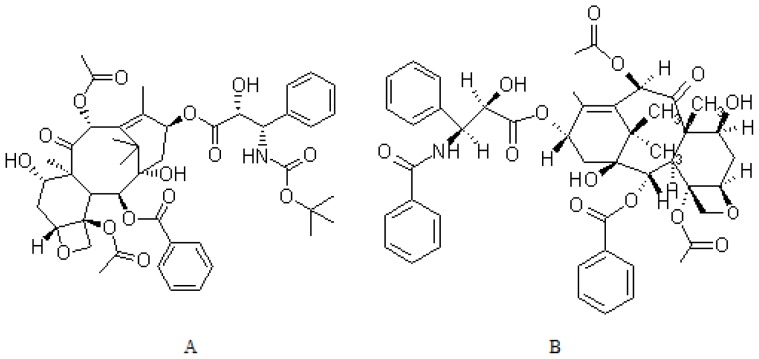
Structure of Docetaxel (DTX) (**A**) and Paclitaxel (**B**).

Docetaxel (Taxotere^®^) (DTX, Figure 1) is a second-generation taxane derived from the needles of the European yew tree, *Taxus baccata* [1], which comprises the most commonly used chemotherapeutic agents for treating solid tumors, especially lung cancer [2,3]. DTX acts by disrupting the microtubular network that is essential for mitotic and interphase cellular functions. It promotes the assembly of tubulin into stable microtubules and inhibits their disassembly, causing inhibition of cell division and eventual cell death. DTX is about twice as potent as paclitaxel in inhibiting microtubule depolymerisation, which has the unique ability to alter certain classes of microtubules [4,5]. Due to its low solubility in water (10 mg/L), it is clinically administered dissolved in high concentration of Tween-80. The efficacy of DTX is frequently limited by their inability to reach the target site of action, especially when DTX is administered through conventional dosage forms or drug delivery systems. The conventional formulations may result in dose-limiting neutropenia, fluid retention, myalgia, neuropathy, hypersensitivity reaction [6], and require extensive premedication, and are responsible for most of the acute toxicity [7]. Then those obstacles limit DTX’s use as a drug despite its reported prominent activity.

Targeted drug delivery systems have increased the amount of drug reaching the site and simultaneously decrease the amount being distributed to other parts of the body. In order to eliminate the Tween-80 based vehicle and in the attempt to increase the drug solubility, alternative dosage forms have been suggested including liposomes [8,9], nanoparticles [10] and micelles [11].

Microspheres technology has been utilized extensively to develop formulations with a sustained release of one therapeutic agent to maintain targeted concentration in the body for a sustained period of time [12]. This drug delivery system has emerged as a remedial measure to improve site-specific drug delivery to a considerable extent and has already been applied to improve the therapeutic response and to reduce adverse effects [13]. The drugs in implant microspheres are absorbed by the capillaries of the injection site and lymph organs, enter the systemic circulation, and then tend to be distributed to the target organ to play a pharmacodynamics [14], which can bypass the first pass effect and avoid pre-systemic elimination in the Gl tract or liver with oral administration.

Chitosan has well-defined properties including bioavailability, biocompatibility and the ability to open the intracellular tight junction; therefore, it has been suggested as a suitable polymeric material for sustained release delivery [15,16]. In this study, we developed chitosan-loaded microspheres for intravenous administration of DTX in order to improve its ability of lung targeting effect. Polymeric microspheres were prepared by an “emulsion crosslinking method” using as matrix chitosan at medium molecular weights [17]. The systems were characterized for surface morphology and size distribution. Technological studies were performed to evaluate the drug, drug-loading coefficient, encapsulation ratio and *in vitro* release. Moreover, pharmacokinetics and biodistribution studies of DTX-loaded chitosan microspheres were investigated.

## 2. Results and Discussion

### 2.1. Preparation and Microsphere Characterization

**Figure 2.**
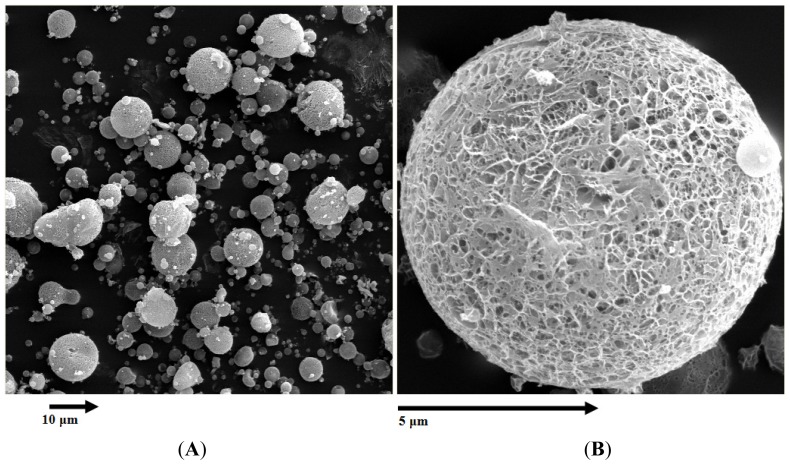
Transmission electron microscope photograph of DTX-loaded microspheres. (**A**) ×300; (**B**) ×3000, (amplification).

As shown in Figure 2, the surface morphology of DTX microspheres was observed by TEM (transmission electron microscope). The microspheres were spherical in shape with a smooth surface and the size was uniform and appropriate for administration via intravenous injection. The encapsulation efficiency and drug loading of prepared microspheres were 88.1% ± 3.5% and 18.7% ± 1.2% (*n* = 5) respectively, and the mean diameter was 9.6 ± 0.8 μm from five batches. The poly disperse index (PDI) was 0.147 ± 0.042 and the zeta potential was +0.26 ± 0.04 mV.

Previous reports have shown that several methods have been used for preparing loaded chitosan microspheres, such as interaction with the anions method [18], the emulsion crosslinking method [19], the precipitation or coacervation method [20], the solvent evaporation method [21], and the spray drying method [22]. In this study, water insoluble reagents (DTX) can be simply dispersed in chitosan solution and entrapped by the emulsion crosslinking process. The method is simple and easy to reproduce with high encapsulation efficiency and ideal drug loading. Meanwhile glutaraldehyde was used as crosslinking agents for the preparation of microspheres.

Microspheres are often prepared using PLGA (poly(lactic-*co*-glycolic acid)) as a polymer. In the present study, however, chitosan was selected as the polymer that was used to prepare DTX microspheres. This was because the DTX content in the PLGA microspheres was very low (<7.5%, *w*/*w*) in our preliminary studies, when they were prepared according to the water-in-oil-in-water emulsion method [23]. The content could be increased to as high as 18.7% in the present study. The stability data of DTX microspheres showed that during stored at 4 °C or room temperature 25 °C for 3 months, surface morphology and content of DTX had no notable changes (Table 1). However, at 37 °C and RH (relative humidity) 75% the agglutinative phenomenon was observed, the mean diameter increased to 30 μm.

### 2.2. *In Vitro* Drug Release Behavior

**Figure 3.**
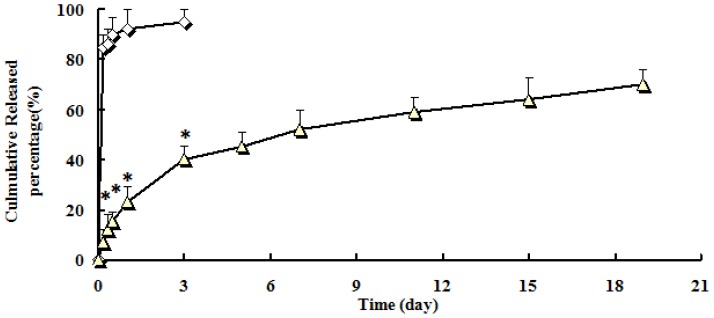
*In vitro* release profiles of DTX-loaded microspheres from three batches. Release experiments were carried out in PBS (phosphate buffer solution) (pH 7.4), at 37 ± 0.5 °C. Each point represents the mean value of three different experiments ± S.D. ⋄: DTX injection; △: DTX-loaded microspheres. * *p* < 0.05: DTX-loaded microspheres (△) *vs.* DTX injection (⋄).

*In vitro* drug release behavior of DTX-loaded microspheres was studied using a shaken method. The release profiles of free DTX and DTX-loaded microspheres were shown in Figure 3. A very fast release behavior of free DTX was observed, whereas the cumulative release rate of DTX-loaded microspheres was much slower followed by a sustained release. In free DTX group, 80% or 94% of DTX were released in the first 12 h (hour) or nineteen days. In contrast, only 23% or 70% of DTX were released from microspheres in the first 12 h or nineteen days (*p* < 0.01). The *in vitro* release was kinetically analyzed according to zero-order, first-order, and the diffusion-controlled release mechanism. The relative high correlation coefficient values obtained from the analysis of the amount of the drug released *vs*. the square root of time indicated the release followed the Higuchi [24] kinetic model. The correlation coefficient (*r*) were as follow: zero order (0.9678); first order (0.9725) and Higuchi (0.9997).

*In vitro* data showed that in comparison with DTX microspheres, the DTX injection released the DTX very fast. In approximately 12 h, 80% of DTX has been released. The results indicated that the DTX microspheres had a well-sustained release efficacy.

The sustained release of DTX from microspheres revealed its applicability as drug delivery system to minimize the exposure of healthy tissues while increasing the accumulation of therapeutic drug in tumor sites.

### 2.3. Pharmacokinetic Studies

**Figure 4.**
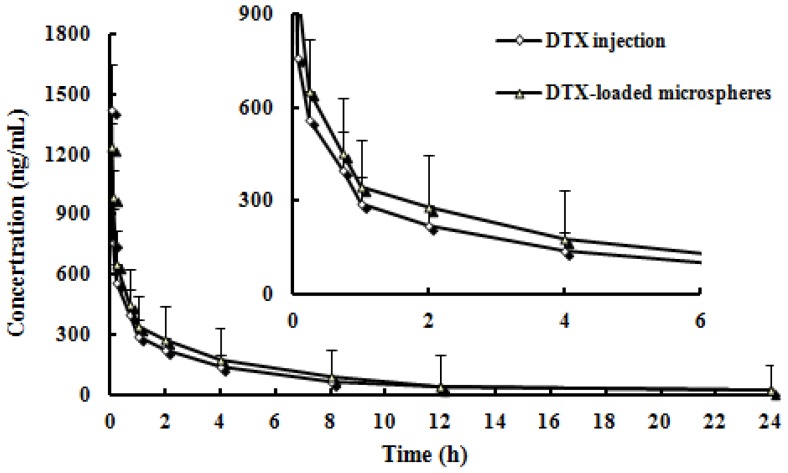
Mean plasma concentration-time profiles of DTX after *i.v.* administration of a single 5 mg/kg dose of injection and microspheres to rats (Each point represents the mean ± SD of 6 rats).

Pharmacokinetic studies were carried out in rats using the microspheres prepared from chitosan. The time course of the plasma concentrations of DTX of injection and microspheres were summarized in Figure 4. The pharmacokinetic parameters calculated from the plasma drug concentration *vs.* time profiles were listed in Table 2. There were significantly different in *t*_1/2α_, *t*_1/2β_, *C*_max_, *AUC*_0–_*_t_*, *AUC*_0–_*_∞_* and *CL* between the DTX-loaded microspheres and DTX injection (*p* < 0.05). Note: *C*_max_, maximum plasma concentration; *AUC*_0–_*_t_*, Area under the concentration–time curve from time 0 to the last measurable concentration; *AUC*_0–_*_∞_*, area under the concentration–time curve extrapolated to infinity; *MRT*, mean residence time; *CL*, plasma clearance.

### 2.4. Biodistribution Studies

**Figure 5.**
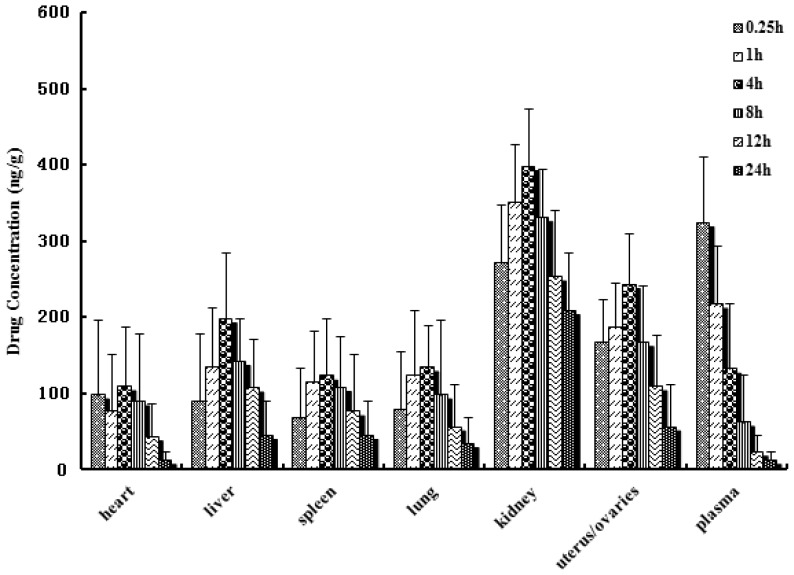
Distribution in tissue in mice after following *i.v.* administration of a single 5 mg/kg dose of DTX injection tissues (ng/g) and plasma (ng/mL), (Each point represents the mean ± SD of 5 mice).

**Figure 6.**
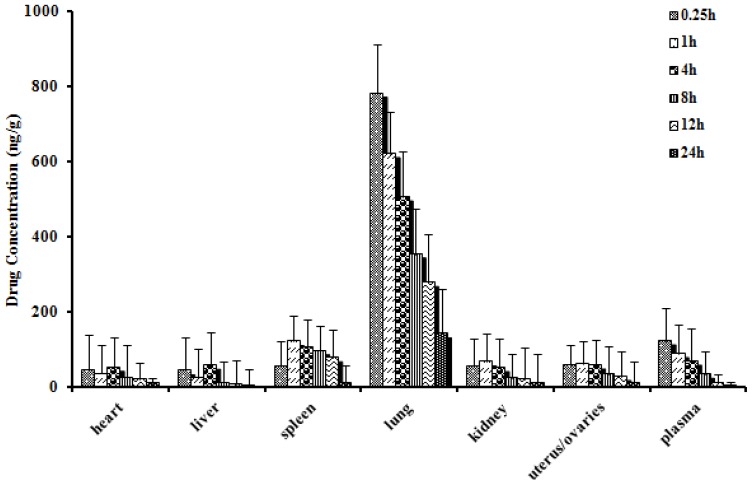
Distribution in tissue in mice after following *i.v.* administration of a single 5 mg/kg dose of DTX-loaded microspheres tissues (ng/g) and plasma (ng/mL), (Each point represents the mean ± SD of 5 mice).

*In vivo* biodistribution behavior of DTX after *i.v.* administration of the DTX-loaded microspheres to mice was investigated with DTX injection as a control. The amounts of drug distributed in unit mass of heart, liver, spleen, lung, kidney, uterus/ovaries and plasma at various times were measured. Figures 5 and 6 present the mean concentration-time profiles of DTX in unit mass of each organ in mice. The total amount of drug accumulated in each organ within 24 h (*AUC*_0–t_) was calculated, and the results are shown in Table 3. The blood DTX concentration-time profiles observed in mice were similar to the pharmacokinetics study in rats. The DTX *AUC*_0–t_ of the microspheres was lower in heart, liver, spleen, kidney, uterus/ovaries and plasma, and higher in lung compared to DTX injection. There were significantly difference in liver, kidney and uterus/ovaries between the DTX-loaded microspheres and DTX injection (*p* < 0.01). No significant difference was observed in spleen, heart and plasma.

From Table 3, one can see that in comparison with DTX injection, DTX microspheres altered the distribution of DTX *in vivo* and the half-life after *i.v.* injection of DTX microspheres (*t*_1/2α_ = 1.31 h, *t*_1/2β_ = 8.6 h) were prolonged remarkably than those (*t*_1/2α_ = 0.24 h, *t*_1/2β_ = 5.2 h) after *i.v.* injection of DTX injection. The result indicated that the DTX microspheres had sustained release efficacy. The drug concentration of tissues was determined by LC-MS/MS (liquid chromatography-mass spectrometry/mass spectrometry). The results indicated that the microspheres could deliver DTX mainly to lung after *i.v.* injection to mice and the concentration of DTX in lung (782.3 ng/g, 0.25 h) was significantly higher than those in other tissue and plasma. Compared with DTX injection, the drug concentration of DTX in lung after *i.v.* injection of DTX microspheres was enhanced from 78.7 to 782.3 ng/g (0.25 h).

In case of targeted drug delivery system such as microspheres, major portion of the drug and excipient are accumulated in specific tissue (lung) and therefore, determining the compatibility between these tissues and the microspheres formulation becomes a necessity in order to estimate the safety of the formulations. In comparison of the microspheres formulations with placebo microspheres, the cytoarchitecture of the tissue (lung) did not show any major difference (degenerative changes). Based on this observation, the constituents of the microsphere formulation were declared safe for parenteral use as a passive targeted drug delivery system to lungs.

The main factor that influences the microspheres for lung targeting is the particle size and surface characterization. The research proved that after intravenous injection, grain size at 7~30 μm can be mechanically intercepted by capillary bed and then accumulate in the lungs. Casettari and others found that positively charged particles and reach lungs easily [25]. The microspheres made by this research had an average grain diameter of 9.6 μm. The carrier material of chitosan was positively charged, which guaranteed the microspheres could have good lung targeting. It can improve the drug concentration in the lungs and reduces the concentration in other tissues, which is propitious to the treatment of lung cancer, and reduces the side effects at the same time.

## 3. Materials and Methods

### 3.1. Materials

DTX was purchased by Hengrui Pharma Co., Ltd. (Lianyungang, Jiangsu, China). Taxotere was obtained from Sanofi Pharma Co., Ltd. (Paris, France). Medium molecular weight chitosan (deacetylation ratio of 75%–85%), glutaraldehyde, paraffin, span-80, mannitol and phosphate buffered saline (PBS) were purchased from Sinopharm, Shanghai, China. Paclitaxel was obtained from Shanghai Institute of Materia Medica, Chinese Academy of Sciences (Shanghai, China). All other materials or solvent were of reagent or analytical grade.

The experiments were performed on rats weighing between 210 ± 20 g and mice weighing between 20 ± 2 g. The animals were kept in cages in a room at a temperature of 25 ± 2 °C, with a 12:12 light-dark cycle. Food and water were freely available. All experiments were performed in strict accordance with the Guide for the Care and Use of Laboratory Animals as adopted by the China National Institutes of Health (Shanghai, China).

### 3.2. Preparation of the Microspheres

A water in oil (W/O) emulsion crosslinking method was used to prepare DTX-loaded chitosan microspheres and the method was simple and easy to scale up [26,27]. In the process, chitosan solution was first prepared by dissolving chitosan with acetic acid. This solution is then added to liquid paraffin containing a surfactant, forming a water-in-oil emulsion before the addition of a crosslinking agent. The formed microspheres were filtered, washed with suitable solvent, and dried. Briefly, About 250 mg chitosan and 40 mg DTX were added to 1 mL of a mixture of 3% acetic acid:ethanol (3:2, *v*/*v*). After completely dissolved, the solution was then slowly added to liquid paraffin (20 mL) containing a surfactant (0.5 g span-80) and homogenized at high stir rates, forming a water-in-oil emulsion before the addition of a crosslinking agent. Then 25% glutaraldehyde solution was slowly added to the emulsion system and crosslinked for 3 h until the microspheres were solidified. The microspheres were collected by filtration through a 20 μm sieve, and then washed three times with cold water. After 1 mL of aqueous mannitol (20%, *w*/*v*) was added to prevent the aggregation of microparticles, the microspheres were freeze dried.

### 3.3. Morphological Characterization and Particle Sizing

The morphological examination of the microspheres was performed using a transmission electron microscope (TEM) (Philips CM120, Amsterdam, The Netherlands). In practice, a drop of microspheres solution containing 0.1% (*w*/*v*) phosphotungstic acids was placed on a carbon film coated on a copper grid and observed at 80 kV in the electron microscope.

Particle size distribution and mean diameter of the prepared DTX-loaded microspheres were determined by dynamic light scattering (DLS, Malvern, Worcestershire, UK) using a NICOMP 380 Submicron Particle Sizer (Santa Barbara, CA, USA) equipped with a 5 mW heliumneon laser at 632.8 nm. Sample solutions were transferred into the light scattering cells. The intensity autocorrelation was measured at a scattering angle of 90° at room temperature. Data was analyzed in terms of intensity-weighted NICOMP distributions (PSS, Jacksonville, FL, USA). Each reported experimental result is the average of at least three *d*_h_ values obtained from analysis of the autocorrelation function accumulated for at least 20 min. Zeta potential was measured on the same samples prepared for size analysis. Note: *d*_h_, diameter of hydromechanics.

### 3.4. Encapsulation Efficiency

The encapsulation efficiency of DTX was determined by dissolving 40 mg of microspheres in 100 μL 3% acetic acid in a 3 mL screw cap (Teflon, Hongchangxin, Beijing, China) tube. 900 μL of 3% acetic acid: acetonitrile (4:5, *v*/*v*) were added and the tubes were shaken vigorously for 30 s. The contents were then allowed to settle for 30 min resulting in a phase separation of approximately 0.5 mL (upper phase, DTX-rich) and 0.5 mL (lower phase). Both phases were analyzed by HPLC methods to determine the amount of DTX encapsulated in the microsphere sample. This method allows for total dissolution of microsphere components and has been shown to results in complete drug partitioning into the upper phase.

### 3.5. Characterization of Microspheres

Drug-loading coefficient (DL%) and encapsulation ratio (ER%) were calculated as described earlier [28,29]. Firstly, DTX was extracted from the microspheres with 1 mL 3% acetic acid: acetonitrile (4:5, *v*/*v*), and then the extract solution was properly diluted prior to HPLC analysis. The content of DTX in the microspheres was determined by HPLC method described below. Then, DL% and ER% were calculated according to Equations (1) and (2):

(1) $$DL\%=W_{\text{M}}/(W_{\text{P}}+W_{\text{M}})\times 100\%$$

(2) $$ER\%=W_{\text{M}}/W_{\text{F}}\times 100\%$$

where *W*_P_ is the weight of initial feeding polymer, *W*_M_ is the weight of drug incorporated in microspheres, and *W*_F_ is the weight of initial feeding drug.

### 3.6. *In Vitro* Drug Release Studies

The *in vitro* release of microspheres was measured in phosphate buffered saline (PBS, pH 7.4) at the temperature of 37 ± 0.5 °C. In a 15 mL centrifuge tube, approximately 150 mg of microspheres were suspended in 6 mL of PBS and shaken horizontally at 100 rpm in a shaking bath maintained at 37 °C. Samples of 2 mL were removed from the tubes at sampling times of 4, 8, 12 h, 1, 3, 5, 7, 11, 15 and 19 days after centrifugation at 4000 rpm for 5 min. The medium removed from the tubes was replaced with the same amount of fresh buffer solution. The collected supernatants were extracted with 1 mL of dichloromethane. The extraction solvent was evaporated and DTX residue was solubilized in 500 μL of acetonitrile. The redissolve samples were subjected to further HPLC analysis.

### 3.7. Pharmacokinetics Evaluation

Twelve rats were used to investigate the effect of microspheres formulation on the pharmacokinetics of DTX after *i.v.* administration. Rats were divided into 2 groups at random and given a single 5 mg/kg dose of the DTX-loaded microspheres and DTX injection by vein injection. Blood samples (0.5 mL) were collected into heparinized tubes from the caudal vein at 2, 5, 15, 45 min and 1, 2, 4, 8, 12 h after *i.v.* administration. Blood was immediately processed for plasma by centrifugation at 2000× *g* for 10 min. Plasma samples were frozen and maintained at −70 °C until analysis.

### 3.8. Biodistribution Studies

Sixty Kunming strain mice were used in the experiment to assess the effect of microspheres formulation on the biodistribution of DTX after *i.v.* administration. The mice were divided into 2 groups at random and given a single 5 mg/kg dose of either the DTX microspheres or DTX injection by tail-vein injection. At 0.25, 1, 4, 8, 12 and 24 h after drug injection, each animal (*n* = 5 for each time point) was euthanized, and heart, spleen, lung, liver, kidney, uterus/ovaries as well as blood samples were collected. Tissue samples were washed in ice-cold saline, blotted with paper towel to remove excess fluid, weighed and stored at −70 °C until assessed for drug concentration by LC-MS/MS.

### 3.9. LC-MS/MS Analysis

The LC-MS/MS experiments were conducted with an API4000 tandem mass spectrometer (Applied Biosystems, Shanghai, China) equipped with a binary and a quaternary pump (Agilent series 1200, Shanghai, China). Analyses were performed on a 5-μm Gemini C_18_ column (50 mm × 2 mm) at a flow-rate of 0.3 mL/min. The mobile phase was methanol:0.1% formic acid solution = 90:10 (*v*/*v*) and the column temperature was maintained at 30 °C. LC-MS/MS was carried out using nitrogen to assist nebulization. The multiple reaction monitoring (MRM) transitions are 830.1→549.0 for DTX; 876.2→591.1 for paclitaxel (internal standard, I.S.). The main working parameters of the mass spectrometer were summarized in Table 4.

50 μL volume of the plasma sample was transferred to a 5 mL plastic test tube together with 10 μL of I.S. solution (10 μg/mL). After vortex shaking for 1 min (5432 vortex mixer, Eppendorf, Germany), 1 mL of MTBE (methyl tertiary butyl ether) was added and the mixture was vortexed for 3 min. After centrifugation at 2000× *g* for 10 min (Thermo IEC, Micromax, Boston, MA, USA), the upper organic layer was quantitatively transferred to a 10-mL glass tube and evaporated to dryness using evaporator at 50 °C. The residue was reconstituted in 100 μL of the mobile phase, and then vortex-mixed. After centrifugation at 1000× *g* for 5 min, 10 μL aliquot of the solution was injected into the LC-MS/MS system for analysis.

Tissue samples were homogenized in a mixed solution of acetonitrile and water (50:50, *v*/*v*). 10 μL of I.S. solution (10 μg/mL) was added into 200 μL of tissue samples, and vortexed for 1 min. The drug and internal standard were then extracted into 3 mL of MTBE by vortex mixing for 3 min. After centrifugation at 6000× *g* for 10 min, the clear supernatant was removed and evaporated under a gentle stream of nitrogen. The residue was then dissolved by 100 μL of the mobile phase, and centrifuged at 1000× *g* for 5 min, 10 μL aliquot of the solution was injected into the LC-MS/MS system for analysis.

### 3.10. Statistical Analysis

Values were expressed as mean ± standard deviation (S.D.) for each group. Statistical evaluation of the experimental data was performed using Student’s *t*-test. A (*p* < 0.05 or *p* < 0.01) was considered statistically significant.

## 4. Conclusions

Docetaxel-loaded, glutaraldehyde-crosslinked chitosan microspheres were prepared using a water-in-oil emulsification method. The prepared microspheres were found to possess suitable physico-chemical properties and the particle size range. The microspheres were found to release the drug to a maximum extent in the target tissue. This work adds to the already significant domain of targeted drug delivery systems, which holds a promising alternative over the conventional means.

## Figures and Tables

**Table 1.** Stability data of DTX-loaded microspheres over three months.

Time (month)	4 °C		25 °C	
	
	Drug remaining (%)	Surface morphology	Drug remaining (%)	Surface morphology
Initial	99.6	spherical	101.3	spherical
1	100.1	spherical	101.7	spherical
2	101.2	spherical	99.7	spherical
3	99.2	spherical	98.9	spherical

**Table 2.** Pharmacokinetic parameters of the two formulations.

Parameter	Formulations	

	Injection	Microspheres
*t*_1/2α_ (h)	0.22 ± 0.1	0.86 ± 0.3 *
*t*_1/2β_ (h)	4.9 ± 1.3	7.9 ± 3.4 *
*C*_max_ (ng/mL)	1425.6 ± 154.6	1239.5 ± 136.3
*AUC*_0–_*_t_* (ng·h/mL)	2234.6 ± 234.1	2555.6 ± 309.2
*AUC*_0–_*_∞_* (ng·h/mL)	2635.8 ± 253.2	2875.3 ± 339.6
*MRT* (h)	12.8 ± 4.6	21.6 ± 3.9 *
*CL* (L/h)	25.8 ± 6.5	16.7 ± 4.2 *

* *p* < 0.05: DTX-loaded microspheres *vs.* DTX injection.

**Table 3.** The *AUC*_0–24 h_ of DTX in plasma and tissues after *i.v.* administration of injection and microspheres to mice (*n* = 5).

Formulation	Heart	Liver	Spleen	Lung	Kidney	Uterus/ovaries	Plasma
DTX injection (ng·h/g)	1332.8	2671.6	1979.4	1772.9	6754.8	3139.8	1494.4
DTX-loaded microspheres (ng·h/g)	613.1	376.3	1699.4	7720.6	651.9	776.1	698.5
Ratio a	0.46	0.14 *	0.86	4.35 *	0.10 *	0.25 *	0.47

a The ratio was AUC (DTX-loaded microspheres)/AUC (DTX injection);

* *p* < 0.01: DTX-loaded microspheres *vs.* DTX injection.

**Table 4.** Main working parameters for tandem mass spectrometry.

Parameter	Value
Scan type	MRM (multiple reaction monitoring)
Ion polarity	Positive
Fragmentor voltage, V	115
Nebulizer pressure, psi	35
Drying gas temperature, °C	340
Dry gas flow, L/min	32
Dwell time per transition, ms	220
Resolution	Unit
Collision energy, eV	30
